# The Influence of High Hydrostatic Pressure on Selected Quality Features of Cold-Storage Pork Semimembranosus Muscle

**DOI:** 10.3390/foods13132089

**Published:** 2024-07-01

**Authors:** Paulina Duma-Kocan, Mariusz Rudy, Marian Gil, Jagoda Żurek, Renata Stanisławczyk, Anna Krajewska, Dariusz Dziki

**Affiliations:** 1 Department of Agricultural Processing and Commodity Science, Institute of Food and Nutrition Technology, College of Natural Sciences, University of Rzeszow, St. Zelwerowicza 4, 35-601 Rzeszów, Poland; pduma@ur.edu.pl (P.D.-K.); mgil@ur.edu.pl (M.G.); rstanislawczyk@ur.edu.pl (R.S.); 2 Department of Financial Markets and Public Finance, Institute of Economics and Finance, College of Social Sciences, University of Rzeszow, Ćwiklinskiej 2, 35-601 Rzeszów, Poland; jzurek@ur.edu.pl; 3 Department of Thermal Technology and Food Process Engineering, University of Life Sciences in Lublin, 31 Głęboka Street, 20-612 Lublin, Poland; anna.krajewska@up.lublin.pl

**Keywords:** meat, high hydrostatic pressure, physicochemical and microbiological properties, sensory analysis

## Abstract

The primary objective of this investigation was to assess the influence of high hydrostatic pressure (HHP) and the duration of cold storage on the physicochemical, technological, and sensory attributes as well as the nutritional composition and shelf life of meat. The experimental framework involved utilizing samples derived from the semimembranosus muscle of pork. Each muscle obtained from the same carcass was segmented into six distinct parts, with three designated as control specimens (K) and the remaining subjected to vacuum packaging and subsequent exposure to high hydrostatic pressure (200 MPa at 20 °C for 30 min). Comprehensive laboratory analyses of the meat were conducted at 1, 7, and 10 days post slaughter. The meat was cold-stored at +3 ± 0.5 °C. The findings of the study elucidated that the application of high hydrostatic pressure exhibited a favorable impact on the extension of the raw meat’s shelf life. The tests showed a significant (*p* < 0.05) decrease in the total number of microorganisms compared to the control sample after 7 (K: 4.09 × 10^5^, HHP: 2.88 × 10^5^ CFU/g) and 10 (K: 7.40 × 10^5^, HHP: 2.42 × 10^5^ CFU/g) days of cold storage. It was also found that using HHP increased the pH value after 1 (K: 5.54, HHP: 5.77) and 7 (K: 5.60, HHP: 5.87) days of storage.

## 1. Introduction

Developing high-quality pork meat and its products is possible with careful consideration of many factors in production. These occur at all stages of livestock and meat production and significantly affect the quality of final products. Increasingly, consumers are expressing a heightened preference for minimally processed food products that possess concomitant attributes of nutritional value, organoleptic satisfaction, and absence of microbiological contamination. In addition to the traditional physical methods of preserving meat and processed foods, i.e., freezing, pasteurization, or sterilization, several new methods have emerged that include high-pressure technique, ultrasound, pulsed light jet, pulsed electric and magnetic fields, low-temperature plasma technique, and vacuum packaging combined with shrinking [1,2,3].

High-pressure technology is an innovative technology that applies to meat preservation and does not usually cause adverse quality changes. High pressure (HP), alternatively known as high hydrostatic pressure (HHP) or high-pressure processing (HPP), is a technological approach that entails placing a food commodity within a pressure chamber, where it undergoes exposure to hydrostatic pressure ranging from 100 to 1000 MPa over a specified duration, typically lasting several minutes. The free space in the chamber is filled with liquid, which transfers the pressure directly to the product. As a result, the distance between molecules is reduced, and their interactions occur [3,4].

The HPP method not only preserves food by destroying harmful microorganisms but also affects the product’s functional properties and sensory characteristics without negatively affecting its nutrients [5,6,7,8]. Nevertheless, beyond its primary function of food preservation, high-pressure technology exhibits an influence on the textural properties of food and has been recognized as a physical process with applicability to the softening (tenderization) of meat and meat products without the incorporation of additional additives [9,10]. The paleness associated with HPP application limits its use in fresh meat. More specifically, more significant pressure leads to protein denaturation and causes gelling of raw meat. Moreover, the use of HHP changes the properties of muscle proteins. They undergo physicochemical changes, i.e., denaturation, dissociation, solubilization, aggregation, and gelation, the rate and extent of which depend on the pressure level, temperature, pH, and ionic strength. Pressure-induced denaturation of proteins involves rearrangement and/or the disruption of non-covalent bonds stabilizing the native structure, such as hydrophobic interaction, hydrogen, and ionic bonds [11]. Sarcoplasm proteins under pressure above 200 MPa are highly susceptible to denaturation as the water-holding capacity and color of the flesh change [12,13]. The process by which microorganisms undergo destruction is attributed to the disruption of cellular functions in living organisms induced by high pressure. This phenomenon leads to unfavorable biochemical alterations, culminating in irreversible modifications to both the cell membrane and spore forms [3]. HPP has been shown to effectively reduce microbial populations in a wide range of food products without requiring high temperatures or chemical additives [14,15]. By adjusting the appropriate parameters of the high-pressure process, it is possible to inactivate both vegetative and spore forms [16,17]. This method can also modify the functional properties of the raw material components and the final product, thereby creating new rheological properties [6]. A prerequisite for the suitability and potential use of high-pressure technology for food preservation is the establishment of such parameters of the compression process that do not reduce the product’s nutritional value and sensory characteristics and preserve its quality and shelf life. Advantages of the high-pressure method include increased product shelf life due to microbial destruction, uniformity of pressure throughout the volume, short processing time, and low energy requirements. The same amount of energy is used regardless of the production batch size [18,19].

Rajendran et al. [20] demonstrated that an application of HHP exceeding 400 MPa induces deleterious alterations in meat quality. Furthermore, Ma and Leward [21] found that as the pressure of 200–800 MPa increased at 20–40 °C, the hardness of beef muscles increased. It was also proved that changes in the color of pork subjected to 200–800 MPa pressure at 5 and 20 °C for 10 min depended mainly on the amount of pressure but to a lesser extent on temperature [22]. Pietrzak et al. [6,23] demonstrated that the applied pressure of 500 MPa for 10 min had no effect on texture, color, or lipid oxidation rate, while a significant reduction in mesophilic microorganisms, psychrophilic microorganisms, and lactic acid bacteria was observed in poultry meat chops. Ros-Polski et al. [24] showed that a lower concentration of NaCl along with HHP treatment had a beneficial effect on the color and texture of poultry meat.

Although there have been many reports on the use of HHP in meat and meat products, many of them concerned pressures in the range of 300–600 MPa [25,26,27,28]. The study of the influence of HHP of 200 MPa at 20 °C for 30 min on the quality of raw pork has not been performed so far. Therefore, the purpose of this study was to assess the impact of high hydrostatic pressure and the duration of cold storage on the physicochemical, microbiological, sensory properties, nutritional composition, and shelf life of the semimembranosus muscle. This would prove the usefulness of this method in extending the shelf life of raw pork meat without negatively affecting most of its quality characteristics.

## 2. Materials and Methods

### 2.1. Material

The material utilized for examination in this research was derived from muscular tissue samples extracted from the semimembranosus muscle (m. semimembranosus) of ham, sourced from pork carcasses acquired from individual farmers associated with producer groups holding contractual agreements with a meat producer located in the southeastern region of Poland. Uniform environmental conditions were maintained for the fatteners subjected to identical complete feeding mixtures. The animals were provided unrestricted access to water. The research encompassed meat samples obtained from 50 fatteners, specifically hybrids resulting from the crossbreeding of female Polska Biała Zwisłoucha and male Duroc breeds, with an equal distribution of 50% gilts and 50% hogs. The carcass weight of the fatteners subjected to slaughter ranged from 110 kg to 120 kg. Adhering to a standard protocol within the meat industry, the fatteners underwent electrical stunning prior to the slaughtering process. Subsequently, following approximately 5 h of confinement in livestock stores post transportation, the slaughtering procedure was executed. The ham muscle, derived from the right half-carcasses, was then excised after a 24 h period of cold storage at +3 ± 0.5 °C.

### 2.2. HHP Meat Processing

The segmentation of each muscle from the identical carcass yielded six distinct parts, wherein three parts were designated as control samples (K), while the remaining three were subjected to vacuum packaging using PA/PE multilayer film using an Inauen Type VC999 vacuum packer (Inauen, Herisau, Switzerland).

**Figure 1:**
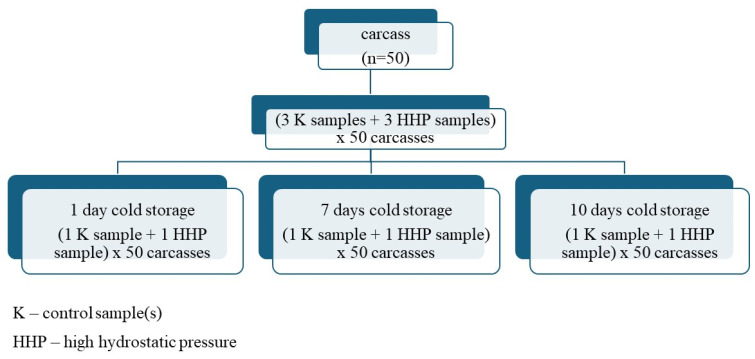
Experiment size diagram of the effect of HHP on the properties of the semimembranosus muscle.

In accordance with the literature findings, subjecting meat to HHP exceeding 400 MPa can induce adverse alterations in various quality attributes, including color, texture, lipid oxidation, and storage integrity [20]. In order to reduce the negative changes in meat, a pressure of 200 MPa was administered at a temperature of 20 °C for 30 min. Three samples from one muscle from one carcass were packaged separately and together exposed to HHP. The number of samples subjected to high pressure at the same time was limited by the capacity of the high-pressure chamber and amounted to 30 samples. Therefore, a total of 5 batches with 30 samples each were utilized. The experiment size diagram is shown in Figure 1. The weight of each sample subjected to HHP was 350 ± 30 g. The pressure build-up time was 100 s, the pressure drop time was 2–4 s, and the time the indicated pressure was obtained and the decompression time are not included in the pressurization times. The pressurization process was carried out at the Institute of High Pressure Physics of the Polish Academy of Sciences in Warsaw, using a U 4000/65 pressure apparatus (Unipress, designed and manufactured by the High Pressure Laboratory), designed with a maximum operating pressure capability of 600 MPa. Two hours after the pressure treatment, the film on the meat samples was cut. Comprehensive laboratory assessments of the meat were conducted after cold-storage periods of 1, 7, and 10 days, with the refrigeration maintained at +3 ± 0.5 °C. Starting from the eighth day onwards, a systematic daily monitoring protocol was implemented to evaluate the freshness of the meat, considering that conventionally stored raw meat without packaging typically manifests a shelf life of approximately seven days post slaughter. Detection of an undesirable (ammoniacal) odor and/or the presence of a slimy surface prompted the removal of the respective meat sample from further cold storage and analysis.

### 2.3. Methods

#### 2.3.1. Chemical Composition

Preceding the assessment of chemical composition, the samples were ground in a laboratory wolf device (Royal Catering, RCMM-2000, Butzbach, Germany) employing a mesh featuring a 4 mm aperture diameter.

The assessment of water content adhered to the guidelines outlined in PN-ISO 1442:2000 [29]. Protein content determination followed the specifications detailed in PN-75/A-04018:2002 [30]. Fat content analysis conformed to the standards stipulated in PN-ISO 1444:2000 [31]. The quantification of salt content adhered to the procedures outlined in PN-A-82112:1973 + Az 1:2002 [32]. Mineral content, expressed as ash, was determined in accordance with the methodologies delineated in PN-ISO 936:2000 [33].

#### 2.3.2. Physicochemical and Microbiological Properties

The pH measurement was conducted employing a CPC-411 pH meter (ELMETRON, Zabrze, Poland) with automatic temperature compensation equipped with an OSH 12-01 electrode, ensuring an accuracy of 0.01. The pH meter was calibrated with pH 4.00 and 7.00 buffers at 20 °C. pH was measured immediately after color determination.

Water activity (aw) was determined utilizing a LabMaster water activity measuring apparatus—aw (Novasina AG, Lachen, SZ, Switzerland).

The oxidation-reduction potential (EH, mV) was estimated employing an ERPt-13-type combination electrode and a waterproof pH/conductometer, specifically the ELMETRON CPC-505 model (Zabrze, Poland).

The assessment of the TBARS index involved the determination of a class of compounds capable of forming colored complexes, particularly with 2-thiobarbituric acid, which includes malondialdehyde. The procedure relies on generating vividly colored complexes formed by aldehydes present in the fat, reacting with a solution of 2-thiobarbituric acid under elevated temperatures. The resultant coloration of the solution derived from the meat product and 2-thiobarbituric acid was quantitatively measured spectrophotometrically (Spekol 2000—Analytik Jena AG, Kundendienst) at 532 nm. The control sample, as outlined by Pikul, Leszczyński, and Kumerow [34], served as the reference solution.

The amount of thermal drip was determined using Janicki and Walczak’s method [35].

Forced meat drip was assessed using the method of Grau and Hamm [36].

The total number of microorganisms adhered to the procedures specified in PN-EN ISO 4833:2004 [37].

#### 2.3.3. Color Measurement

Sample surfaces were cut and allowed to bloom for a 30 min prior to color evaluation. Instrumental color measurement in the CIE L*a*b* system was performed on a meat cross-section using a HunterLab UltraScan PRO electronic colorimeter (HunterLab, Reston, VA, USA), illuminant D65, measuring head opening 20 mm. The electronic colorimeter was calibrated using a white standard: L*—99.18; a*—0.07; b*—0.05. Sample surfaces were cut and allowed to bloom for a 30 min, and then, the color was measured. Instrumental determinations were made as previously described using a HunterLab electronic colorimeter.

The browning index (BI) was determined according to Pérez-López et al. [38].

The total color difference (∆E) between the control and HHP-treated samples was calculated based on the color results. The calculation of the total color difference followed the methodology outlined by Cserhalmi et al. [39].

The determination of heme dye content involved an extraction process utilizing cold phosphate buffer (0.04 M, pH 6.8), followed by clarification through centrifugation (at +4 °C) for 60 min at 15,000 rpm. Following clarification, absorbance measurements were recorded at wavelengths of 525 nm, 545 nm, 565 nm, and 572 nm using the Spekol 2000 spectrophotometer (Analytik Jena AG, Kundendienst). The reference solution utilized for these measurements comprised phosphate buffer, as prescribed by Krzywicki [40].

#### 2.3.4. Texture Parameters Measurement

The evaluation of texture parameters adhered to established methodologies outlined in the literature, specifically as documented by Jaico et al. [41] and Ma and Ledward [21]. Instrumental texture assessment was conducted on meat samples, cut into cube shapes with a side length of 20 mm, and affixed with a cylindrical attachment. A 2-fold compression test was applied to the samples, compressing them to 50% of their original height. The cylinder’s testing speed was 2 mm/s, with a 2 s interval between successive pressures. Texture parameters were ascertained using the Texture Pro CT software v. 1.9 software (Brookfield, WI, USA).

The average value of six consecutive repetitions was taken as each trial’s final texture parameter measurement.

#### 2.3.5. Sensory Evaluation

Sensory analysis was conducted according to method described by Baryłko-Pikielna and Matuszewska [42]. A sample of meat (100 g) underwent heat treatment in a water bath Funke Gerber WB436-D (Berlin, Germany) at a temperature of 100 °C. The heat treatment continued until the internal temperature of the meat reached 80 ± 2 °C. The temperature was monitored using a Sous Vide Thermapen thermometer (MERA, Warsaw, Poland) equipped with a needle probe. Subsequently, the heat-treated samples were cooled to 20 ± 2 °C and sliced into 1.5 cm thick sections perpendicular to the muscle fibers for sensory assessment. All samples were placed in covered plastic containers marked with unique digital codes. The sensory evaluation was performed at room temperature in individual booths with white light on the day. The selection of samples for sensory evaluation was conducted in a random order. The sensory evaluations were performed by a permanent laboratory team comprising eight panelists, each possessing expertise in meat and processed meat product evaluation. The team adhered to the principles outlined in ISO 8586-2 [43] and ISO 8587 [44], focusing on sensitivity and sensory performance. The evaluation team comprised individuals aged between 30 and 55, with an equal distribution of 50% male and female participants. The evaluators were adept in assessing the sensory attributes of meat and processed meat products. The sensory evaluation employed a 5-point partial quality scale assessing the following quality indicators: aroma intensity (1 = very negative, very weakly perceptible; 5 = very strong), taste intensity (1 = very negative, very weakly perceptible; 5 = very strong), aroma desirability (1 = undesirable; 5 = highly desirable), taste desirability (1 = undesirable; 5 = highly desirable), juiciness (1 = very dry; 5 = very juicy), and tenderness (1 = very tough; 5 = very tender). Before testing each sample, the evaluators took a 30 s break and washed their mouths with mineral water. The evaluation was conducted in 15 sessions, and 20 samples were assessed in each.

### 2.4. Statistical Analysis

All observations composing the experiment (2 treatments × 50 batches × 3 storage periods) were included in the statistical analysis. The data were verified for normality using the Kolmogorov–Smirnov test. The homogeneity of variance was verified using the Brown–Forsythe test. Selected physicochemical and microbiological properties, texture, and sensory evaluations of the semimembranosus muscle were analyzed by a two-way analysis of variance (ANOVA), using the GLM procedure in Statistica (STATISTICA ver. 13.3; Stat Soft, Krakow, Poland), which considers treatment applied or cold-storage time as a fixed effect and batch as a random term. In sensory variables, the model included the batch in addition to the main effects and their interaction as well as the panelist in sensory evaluation, with the batch used as the error term to test the significance of the main effects and their interaction. The post hoc Tukey’s test were used to determine the statistical significance among the means at a *p* < 0.05 significance level. Mean values and standard errors of the means (SEM) (all tables) were also reported. All data analysis was performed using the GLM procedure in Statistica (STATISTICA ver. 13.3; Stat Soft, Krakow, Poland).

## 3. Results and Discussion

### 3.1. Chemical Composition

The chemical composition of meat after cold storage is shown in Table 1.

No significant differences (*p* < 0.05) were observed in the concentrations of the analyzed chemical components after the application of HHP treatment. A marginal elevation in fat content was noted during cold storage in the samples treated with high pressure compared to the control trials. The current scientific literature does not provide specific data concerning the influence of HHP on the chemical composition of meat.

### 3.2. Physicochemical and Microbiological Properties

Table 2 shows the physicochemical and microbiological characteristics of the meat. The pH, thermal properties, and forced drip of the meat indicate its freshness and favorable hydration qualities based on the raw material utilized in the investigation. Examination of the data in Table 2 reveals that the application of HHP resulted in a statistically significant increase (*p* < 0.05) in pH. The study revealed differences between the control group and the group subjected to HHP treatment on the initial day of the cold-storage duration as well as between the control group and the HHP-treated group on the 10th day of the cold-storage period. A two-factor analysis of variance showed that the type of treatment had a statistically significant effect on the differences in meat pH. The increase in meat pH after HPP is a well-known phenomenon [25,27,45]. Similar results were obtained by Cheah and Ledwar [25]. The authors showed an increase in the pH value in ground pork meat stored under cold conditions with applied pressure from 200–800 MPa compared to a control sample. Furthermore, McArde et al. [27] showed an increase in pH values with applied pressures of 400–600 MPa in beef meat compared to a control sample. The increase in pH after HPP is attributed to a reduction in available acid groups in the meat due to conformational changes associated with protein denaturation [45].

The utilization of HHP did not yield statistically significant effects on the quantities of forced or thermal drip in the meat. These values throughout cold storage were at similar levels compared to the control sample. Research conducted by various authors [46,47,48] has demonstrated that the application of higher pressures within the range of 300–400 MPa leads to increased water losses in the analyzed samples compared to pressure within the range of 200 MPa. Water activity, conversely, governs the progression of biological phenomena in meat, encompassing the assessment of microorganism growth [49]. The analysis of the obtained results revealed that the values of water activity, following the application of HHP throughout the entire cold-storage period, remained consistent, ranging from 0.966 to 0.988.

Lipid oxidation stands as a predominant factor contributing to the deterioration of food, particularly evident in meat products [50]. Despite the recognized preservative impact of HPP on meat, it is noteworthy that under elevated pressure conditions, the susceptibility to lipid oxidation is heightened [51]. The TBARS index values identified in the meat subjected to high-pressure treatment exhibited comparable levels to those observed in the control samples, and no statistically significant differences were noted between the analyzed groups over the entire duration of cold storage. Similar values were obtained by McArdle et al. [46]. The authors showed that high hydrostatic pressure at 200 MPa had no significant effect on the TBARS index values of beef. However, a study by Cheah and Ledward [25] found that an increase in the applied pressure of 400–600 MPa increased the TBARS values of ground pork. Also, a study by McArdle et al. [27] showed a trend for higher TBARS values at 600 MPa compared to samples exposed to lower pressures up to 400 MPa. HPP is generally believed to increase the rate of lipid oxidation, especially at pressure levels above 300 MPa [25]. Cheah and Ledward [25] concluded that the rate of lipid oxidation remained unchanged at pressures less than 300 MPa, which is consistent with the pressure level used in the present study. The data in Table 2 showed that after applying HHP, compared to the control sample, the values of oxidation-reduction potential of meat were lower in all periods of cold storage. Statistically significant differences were found between the control group on the 1st day of storage, the control group on the 7th and 10th day of storage, and the HHP-treated group on the 1st and 7th day of cold storage. Moreover, a statistically significant difference was found between the control sample and the sample treated with HHP on the 1st day of cold storage. Furthermore, statistically significant differences were identified between the samples subjected to HHP treatment on the 1st day of cold storage and those treated with HHP on the 7th and 10th day. The type of treatment, storage time, and the interaction effect between storage time and type of treatment had a statistically significant effect on the value of oxidation-reduction potential. The degree of microbial inactivation depends on factors such as the type of microorganism, the amount of pressure, the temperature and time of the process, pH, and components of the food or dispersion environment [52,53]. This investigation demonstrated that the application of HHP led to a reduction in the total microbial count in meat throughout the cold-storage period, with statistically significant differences becoming evident only after 7 and 10 days of storage. Additionally, a two-factor analysis of variance revealed a statistically significant influence of the treatment type on the overall microbial count. Microorganism inactivation is a non-selective process in which the most important mechanism is the photochemical effect. The degree of reduction is determined by the type of meat, its chemical composition, and the type and physiological state of microorganisms [54]. The basic principles of HPP microbial inactivation are based on protein denaturation, which results in enzyme inactivation, and the agglomeration of cellular proteins [55]. The change of the permeability of the cell membrane, however, results from the crystallization of fatty acids from phospholipids [56]. Comparable findings were reported by Kloczko [26], who observed a decline in microbial numbers in pork meat upon applying high pressures at 400 MPa. Similar outcomes were echoed by Sun et al. [57], investigating the impact of HPP processing on the safety and quality of beef steaks intended for sous-vide processing (55 °C for 2 h). Inoculated with *Escherichia coli* 0157: H7, the meats underwent pre-cooking, vacuum packing, and pressurization. HPP treatment at 450 MPa for 15 min resulted in a 4.74 log CFU/g reduction in these bacteria. Conversely, after 10 min at 600 MPa, the reduction in *Escherichia coli* count was 6.13 log CFU/g. Additionally, a study by Lee et al. [28] on beef steaks using HPP above 300 MPa showed significant microbial inactivation. A study by Pietrzak et al. [23] proved that HPP treatment effectively improves the microbiological quality of vacuum-packed poultry meat preparations. Elevated hydrostatic pressure induces microbial inactivation by instigating structural and morphological alterations in bacterial cells. The application of HPP can potentially compromise the integrity of the cell membrane, resulting in diminished membrane fluidity and the denaturation of membrane-bound proteins. It is noteworthy, however, that the resistance of bacterial cells to pressure varies depending on the specific bacterial species and the characteristics of the digestive system [58,59,60].

### 3.3. Instrumental Color

A crucial criterion for the technological quality of meat is its color. The muscle dye accountable for coloration is myoglobin, undergoing oxidation to form either oxymyoglobin or metmyoglobin. Studies indicate that HPP causes extensive changes in the color of fresh meat [61]. The outcomes of the investigation into the impact of high-pressure treatment on the color parameters L*, a*, and b*; browning index (BI); total absolute color difference; and the heme pigment content of meat during cold storage are detailed in Table 3.

The statistical analysis revealed a noteworthy influence of the treatment type on color parameters including L*, a*, and b* and the browning index. Furthermore, a statistically significant impact of storage duration on the overall color content was observed. The application of HHP demonstrated a statistically significant (*p* < 0.05) effect on enhancing color brightness (L*) by approximately 50% across all periods of cold storage, accompanied by an increase in the value of the color parameter b*. This implies that the application of high hydrostatic pressure, despite the reduction in the total number of microorganisms after 7 and 10 days of cold storage, significantly increases the color brightness of meat in all periods of cold storage, making raw meat unattractive to consumers since color is the primary and usually the first criterion consumers consider for purchasing this raw material. A significant (*p* < 0.05) effect of HHP on the color parameter a* at 7 and 10 days of cold storage was also observed. Moreover, it was shown that the application of HHP significantly affected the BI index at 1 and 10 days of storage. The total color difference after the initial day of storage amounted to 18.28. As the storage duration increased, the ∆E exhibited a marginal decrease, reaching 14.37 on the 10th day of cold storage. There was no statistically significant effect of HHP on changes in the content of individual forms concerning heme dyes. However, it should be noted that the (MB) values were reduced after the ten days of cold storage. An increase in L* values is the most commonly reported modification occurring in raw meat processed at or above 200 MPa [62]. Studies conducted by several authors [9,27,61,63,64,65] also found an increase in the brightness component of meat color (L*) in the range of applied pressure of 200 ÷ 350 MPa and a change from red to a brighter pink color. Under high pressure, the value of the yellow color (b*) increased or remained at the same level. It was also shown that the changes in the color of pork treated at 200 ÷ 800 MPa at 5 and 20 ºC for 10 min depended mainly on the amount of pressure and, to a minor extent, on the applied temperature [23]. Also, Cheftel and Culioli [56] showed that the color of beef and pork meat undergoes brightening under high pressure; i.e., it loses its red hue, turning gray-brown. Moreover, a study conducted by McArdle et al. [27] on beef meat showed no statistically significant differences regarding the b* parameter between the groups studied. Carlez et al. [66] similarly observed no alterations in b* values in ground beef exposed to high pressure up to 500 MPa in their investigation. The variations in L* and a* values under high-pressure conditions may be ascribed to myoglobin denaturation, stemming from the disruption of the porphyrin ring or the liberation and modification of components within the sarcoplasmic protein fraction [67]. Furthermore, the color differences can be attributed to pressure-induced changes in the water–protein binding properties. These changes could have caused differences in the way the light was scattered, causing the color to appear lighter or darker. A study conducted by Lee et al. [28] showed a similar trend of decreasing ∆E in beef steaks during cold storage. In general, HPP color-induced changes vary according to the myoglobin content and are more dramatic for fresh red meat than for white meat and cured meat products. Undesired changes can be limited by optimizing the process parameters of HPP treatment, such as pressure, time, temperature, curing, oxygen removal, and increased pH. When looking for a reduction in the color changes induced by HPP, one should keep in mind that measures to protect the color quality and stability can result in changed microbial inactivation kinetics and thus impact the safety and shelf life of the final product [55].

### 3.4. Texture Parameters

Besides its ability to preserve food, high-pressure technology also impacts the texture of food and was identified as a physical process applicable to softening (tenderizing) meat and meat products without additives. The food industry also uses such structural modifications of meat proteins to develop new products. Table 4 shows results for texture parameters of cold-stored meat. The study proved a statistically significant (*p* < 0.05) effect of HHP on the variation of such texture parameters as springiness at 1 and 10 days of storage and rigidity at 5 and 7 days of cold storage. There were no statistically significant differences for the other texture parameters, i.e., cycle 1 and 2 hardness, rigidity 8, cohesiveness, adhesiveness, resilience, and chewiness. It was found that the application of HHP affected the increase in cycle 1 and cycle 2 hardness throughout the cold-storage period. Also, the rigidity of meat increased (*p* < 0.05) after 7 days of cold storage after application of this treatment. After applying HHP, chewiness also increased after 7 and 10 days of cold storage. HHP treatment also increased the springiness of the analyzed muscles on days 7 and 10 of cold storage. However, after 1 day of cold storage, the rigidity 5 and 8 decreased (statistically non-significantly) after application of HHP. In the case of springiness, there was a decrease (*p* < 0.05) in this feature compared to the control sample. This was most likely because all the processes associated with maturation were not completed in the meat during this period compared to the raw material stored for 7 and 10 days. Two-factor analysis of variance showed that the type of treatment had a statistically significant effect on the differences in only selected texture parameters, such as rigidity 5 and springiness.

In a study by Ma and Ledward [21], the authors showed that as the pressure increased from 200 to 800 MPa with temperatures in the range of 20–40 °C, the hardness of beef muscles increased. Kloczko [26] showed that in terms of texture (hardness, tenderness, gumminess, and chewiness), pork samples treated with HPP showed no significant differences from control samples. Pressure causes texture modifications, affecting the structure of myofibrillar proteins and their gel-forming properties. The thermolabile nature of muscle proteins and the effect of pressure on the gelling properties of meat and meat proteins will allow the development of new meat products with improved structure. Economic benefits in the form of time and energy savings are associated with the use of HPP as a processing stage in the meat industry. Meat tenderization by HPP is probably due to the breakdown of lysosomes and the subsequent release of proteolytic activity into the medium [55]. Cheftel and Culioli [56] believe that texture changes result from high-pressure-induced disruption of protein functionality, particularly myosin-heavy chains, which ultimately cause texture changes.

### 3.5. Sensory Characteristic

**Figure 2:**
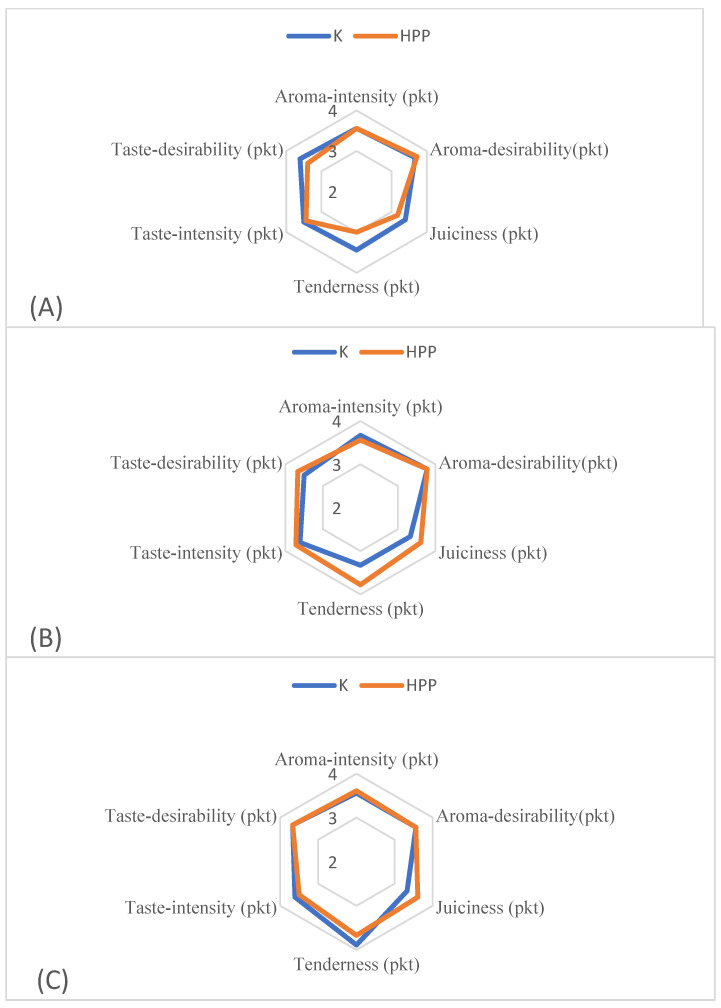
Sensory analysis of meat after cold storage. (**A**) 1 day cold storage; (**B**) 7 days cold storage; (**C**) 10 days cold storage. Explanatory notes: K—control sample; HHP—high hydrostatic pressure (200 MPa—30 min). There were no statistically significant differences between the groups.

Figure 2 illustrates the outcomes of the sensory evaluation of meat following cold storage. The presented data suggest that neither the application of HHP nor the storage duration yielded statistically significant differences in the perception of selected sensory attributes of the meat. Nevertheless, a slightly more favorable assessment of the juiciness and tenderness of HHP-treated meat in subsequent storage days was noted compared to the control sample. On the 7th day of cold storage, the juiciness of raw pork samples subjected to HHP was assessed at 3.61 points and that of the control sample at 3.33 points. A similar relationship was found in the case of the point assessment of tenderness in the tested samples. On the 7th day of cold storage, the tenderness assessment in samples treated with HHP was higher and amounted to 3.78 points compared to the control sample at 3.33 points. Lee et al. [28], employing a higher hydrostatic pressure of 500 MPa, reported enhanced sensory quality in beef steaks compared to the control sample. Additionally, Kloczko [26] demonstrated a positive impact of HHP on the sensory evaluation of pork in their study. The author showed that in terms of cross-sectional appearance, aroma, firmness, and elasticity, higher scores were obtained by samples treated with high hydrostatic pressure. In turn, Kruk et al. [68], analyzing the effect of high pressure on the quality of poultry meat, showed that the technique used negatively affected the sensory characteristics of chicken breast fillets. A pressure of 300 MPa significantly altered the fillets’ taste, aroma, and juiciness, while a pressure of 450 MPa caused the meat to lose its aroma.

## 4. Conclusions

Following the application of high hydrostatic pressure, there was a consistent reduction in the total microbial count compared to the control sample throughout the entire cold-storage period. This suggests that such treatment may serve as an effective means of lowering the total number of microorganism in raw pork. The implementation of high hydrostatic pressure resulted in a statistically significant increase (*p* < 0.05) in both brightness and b* parameter values of meat color across all storage durations, along with an elevation in pH observed on days 1 and 10 of storage. Conversely, high hydrostatic pressure demonstrated no significant impact on the chemical composition, thermal and forced drip, texture, sensory evaluation, and lipid oxidation rate of the examined meat. To minimize the adverse effects of high hydrostatic pressure on some quality characteristics of raw meat (e.g., color brightness), optimizing the combination of time and pressure values in conjunction with other factors or treatments during future studies will be necessary.

## Figures and Tables

**Table 1:** Chemical composition of meat after cold storage.

Specification	Treatments	Cold-Storage Period (Days)			SEM	ANOVA
		1	7	10		
Fat (%)	K	3.05	2.63	2.20	1.23	-
	HHP	3.08	3.37	3.75	1.38	
	SEM	1.25	1.22	1.43		
Water (%)	K	74.92	75.23	75.67	1.16	-
	HHP	74.98	74.77	72.33	1.06	
	SEM	1.08	1.21	5.81		
Protein (%)	K	20.68	20.57	20.77	0.37	-
	HHP	20.78	20.67	19.75	1.52	
	SEM	0.29	0.32	2.24		
Minerals (%)	K	1.42	1.51	1.42	0.22	-
	HHP	1.42	2.04	1.59	0.20	
	SEM	0.17	0.23	0.23		
Salts (%)	K	0.78	0.86	0.69	0.09	-
	HHP	0.78	0.90	0.63	0.11	
	SEM	0.17	0.10	0.04		

Explanatory notes: K—control sample; HHP—high hydrostatic pressure (200 MPa—30 min); SEM—standard error of mean; ANOVA—two-factor analysis of variance between the cold-storage time (T) and type of treatment (Z), *p* < 0.05. There were no statistically significant differences between the groups.

**Table 2:** Physicochemical and microbiological properties of meat after cold storage.

Specification	Treatments	Cold-Storage Period (Days)			SEM	ANOVA
		1	7	10		
pH	K	5.54 ^ax^	5.75 ^ax^	5.60 ^ax^	0.15	Z *
	HHP	5.77 ^ay^	5.70 ^ax^	5.87 ^ay^	0.13	
	SEM	0.05	0.18	0.20		
Water activity	K	0.988	0.969	0.966	0.003	-
	HHP	0.987	0.971	0.971	0.006	
	SEM	0.006	0.003	0.006		
Thermal drip (%)	K	21.12	22.79	20.22	1.58	-
	HHP	23.30	21.52	16.79	2.27	
	SEM	0.91	2.06	2.81		
Forced drip (cm^2^)	K	4.46	2.99	3.73	1.35	-
	HHP	4.49	3.35	2.56	0.50	
	SEM	1.14	1.08	0.59		
TBARS index (mg MDA/kg)	K	0.55	0.58	0.60	0.04	-
	HHP	0.55	0.57	0.57	0.04	
	SEM	0.03	0.03	0.05		
Oxidation-reduction potential (mV)	K	390.90 ^ax^	327.70 ^bx^	304.00 ^bx^	12.51	T *;
	HHP	272.13 ^ay^	318.40 ^bx^	297.83 ^abx^	8.27	Z *;
	SEM	16.15	5.44	9.58		T × Z *
Total number of microorganisms (CFU/g)	K	1.46 × 10^4 ax^	4.09 × 10^5 bx^	7.40 × 10^5 cx^	0.44 × 10^5^	Z *
	HHP	1.27 × 10^4 ax^	2.88 × 10^5 ay^	2.42 × 10^5 ay^	0.61 × 10^5^	
	SEM	0.13 × 10^4^	0.71 × 10^5^	0.8 × 10^5^		

Explanatory notes: K—control sample; HHP—high hydrostatic pressure (200 MPa—30 min); ^a,b,c^—differences in the same rows are statistically significant at the level *p* < 0.05, and no letters or the same letters indicate no statistically significant differences; ^x,y^—differences in the same columns are statistically significant at the level *p* < 0.05, and no letters or the same letters indicate no statistically significant differences; SEM—standard error of mean; ANOVA: two-factor analysis of variance between the cold-storage time (T) and type of treatment (Z), * *p* < 0.05.

**Table 3:** Color parameters, browning index (BI), total color difference (∆E), and heme pigment content of meat after cold storage.

Specification	Treatments	Cold-Storage Period (Days)			SEM	ANOVA
		1	7	10		
L*	K	52.99 ^ax^	51.15 ^ax^	48.80 ^ax^	3.40	Z *
	HHP	71.17 ^ay^	63.45 ^ay^	63.09 ^ay^	5.74	
	SEM	3.65	5.12	4.94		
a*	K	16.95 ^ax^	14.65 ^bx^	14.75 ^bx^	1.56	Z *
	HHP	17.57 ^ax^	16.85 ^ay^	15.73 ^ay^	1.78	
	SEM	2.01	2.17	0.88		
b*	K	9.92 ^ax^	8.20 ^bx^	7.56 ^bx^	1.07	Z *
	HHP	11.70 ^ay^	10.12 ^ay^	8.73 ^ay^	0.88	
	SEM	0.89	1.17	0.88		
BI	K	43.08 ^ax^	37.50 ^bx^	37.91 ^bx^	2.90	Z *
	HHP	35.30 ^ay^	35.99 ^ax^	32.36 ^ay^	2.58	
	SEM	3.11	2.57	2.54		
∆E		18.28	12.64	14.37		
MB (%)	K	46.09	42.16	45.99	13.28	-
	HHP	54.42	46.64	42.66	10.56	
	SEM	12.01	5.88	17.88		
METMB (%)	K	31.78	33.46	28.00	12.51	-
	HHP	20.35	34.99	33.21	8.68	
	SEM	12.35	5.56	13.87		
MBO (%)	K	22.14	24.38	26.00	8.12	-
	HHP	25.23	18.37	24.13	12.90	
	SEM	10.84	8.03	12.68		
OZB (mg/kg)	K	7.35 ^ax^	2.90 ^bx^	6.95 ^ax^	0.89	T *
	HHP	7.26 ^ax^	2.81 ^bx^	7.08 ^ax^	0.72	
	SEM	1.17	0.20	1.08		

Explanatory notes: K—control sample; HHP—high hydrostatic pressure (200 MPa—30 min); MB—myoglobin; METMB—metmyoglobin; MBO—oxymyoglobin; OZB—total dye content; ^a,b^—differences in the same rows are statistically significant at the level *p* < 0.05, and no letters or the same letters indicate no statistically significant differences; ^x,y^—differences in the same columns are statistically significant at the level *p* < 0.05, and no letters or the same letters indicate no statistically significant differences; SEM—standard error of mean; ANOVA—two-factor analysis of variance between the cold-storage time (T) and type of treatment (Z), * *p* < 0.05.

**Table 4:** Texture parameters of meat after cold storage.

Specification	Treatments	Cold-Storage Period (Days)			SEM	ANOVA
		1	7	10		
Hardness 1 (N)	K	210.99	133.75	117.66	48.58	-
	HHP	213.14	158.95	151.61	59.45	
	SEM	43.25	47.40	71.40		
Hardness 2 (N)	K	144.98	88.70	85.80	35.71	-
	HHP	145.30	99.07	101.35	46.51	
	SEM	33.08	41.44	48.82		
Rigidity 5 (N)	K	55.76 ^ax^	11.43 ^bx^	13.80 ^bx^	13.36	Z *
	HHP	37.11 ^ax^	20.47 ^by^	15.55 ^bx^	12.68	
	SEM	23.77	6.63	8.68		
Rigidity 8 (N)	K	156.25	35.00	61.51	28.02	-
	HHP	123.22	44.00	92.15	30.50	
	SEM	48.54	6.00	33.25		
Adhesiveness (mJ)	K	1.88	1.89	2.52	1.19	-
	HHP	2.38	2.24	2.21	1.01	
	SEM	1.26	0.63	1.42		
Cohesiveness	K	0.23	0.24	0.29	0.08	-
	HHP	0.23	0.20	0.29	0.11	
	SEM	0.1	0.08	0.12		
Springiness (mm)	K	5.05 ^ax^	3.17 ^bx^	3.44 ^bx^	0.94	Z *
	HHP	3.67 ^ay^	3.40 ^ax^	4.99 ^by^	0.91	
	SEM	1.16	0.54	1.09		
Resilience	K	0.10	0.20	0.20	0.07	-
	HHP	0.13	0.17	0.15	0.08	
	SEM	0.07	0.08	0.08		
Chewiness (mJ)	K	251.80	96.30	111.42	45.49	-
	HHP	177.17	115.58	202.06	87.60	
	SEM	51.82	60.10	87.72		

Explanatory notes: K—control sample; HHP—high hydrostatic pressure (200 MPa—30 min); ^a,b^—differences in the same rows are statistically significant at the level *p* < 0.05, and no letters or the same letters indicate no statistically significant differences; ^x,y^—differences in the same columns are statistically significant at the level *p* < 0.05, and no letters or the same letters indicate no statistically significant differences; SEM—standard error of mean; ANOVA—two-factor analysis of variance between the cold-storage time (T) and type of treatment (Z), * *p* < 0.05.

## Data Availability

The original contributions presented in the study are included in the article, further inquiries can be directed to the corresponding authors.
